# The Peking Health Anxiety Scale for Infectious Diseases: psychometric properties and short-form development

**DOI:** 10.3389/fpsyt.2025.1734657

**Published:** 2026-01-05

**Authors:** Junkai Feng, Yixuan Wang, Yinyin Zang

**Affiliations:** School of Psychological and Cognitive Sciences and Beijing Key Laboratory of Behavior and Mental Health, and Key Laboratory of Machine Perception (Ministry of Education), Peking University, Beijing, China

**Keywords:** COVID-19, health anxiety, infectious diseases, psychometrics, scale development, screening

## Abstract

**Background:**

Health anxiety can escalate rapidly during infectious disease outbreaks, yet existing assessment tools lack specificity for such contexts. This study aimed to develop and validate the Peking Health Anxiety Scale for Infectious Diseases (PHAID), a brief, context-sensitive measure tailored to infectious disease-related health anxiety.

**Methods:**

The PHAID was adapted from the Short Health Anxiety Inventory, with items revised to focus on COVID-19. Psychometric validation was conducted in a sample of 1,660 adults recruited primarily from the United States via Amazon’s MTurk during the COVID-19 pandemic. Factor structure was determined using exploratory and confirmatory factor analyses. Reliability, convergent and discriminant validity, and test–retest stability were assessed. A clinical cutoff and the relationship with preventive behaviors were examined. A 5-item short form (PHAID-S) was developed using Item Response Theory.

**Results:**

The PHAID demonstrated a stable two-factor structure (Catastrophic Thinking and Infection Worries), excellent internal consistency (*α* = 0.931 and ω = .947), good test–retest reliability (*r* = 0.83), and strong convergent and discriminant validity. PHAID scores showed a cubic relationship with handwashing frequency, distinguishing adaptive vigilance from excessive behaviors. The PHAID-S retained good reliability (α = 0.85) and screening accuracy. The cutoff scores were 24 for PHAID and 11 for PHAID-S.

**Conclusions:**

The PHAID and its short form provide reliable and valid tools for identifying infectious disease-related health anxiety. They show promise for supporting research and public health screening during outbreaks, though further validation in more diverse populations and clinical settings is warranted.

## 1 Introduction

Health anxiety refers to persistent worries and fear about the perceived threats to one’s health (1–3). Conceptualized as a continuum, it ranges from minimal health awareness to excessive anxiety, previously termed hypochondriasis (4). According to the cognitive-behavioral model, this anxiety is maintained by maladaptive processes, including the catastrophic misinterpretation of bodily sensations and safety-seeking behaviors (4–6).

Illness-related events can trigger health anxiety (7), with infectious disease outbreaks such as SARS, H1N1 influenza, and COVID-19 acting as especially potent stressors (8). Several features make infectious diseases particularly anxiety-provoking: their transmission can be covert and unpredictable, often involving aerosols or asymptomatic carriers (9–14). Moreover, the physical symptoms of anxiety (e.g., shortness of breath, chest tightness) can closely mimic those of infectious diseases, leading to misinterpretation of benign sensations (15). Additionally, the perceived severity and lethality of certain infectious diseases can intensify fears of infection and death (16). Together, these factors create a context in which health anxiety can escalate rapidly during infectious disease outbreaks. While severe health anxiety is linked to higher risks of mental disorders (e.g., depression and generalized anxiety; 17, 18), impaired stress coping (19, 20), and reduced quality of life (21), moderate levels can motivate adaptive behaviors during pandemics, such as social distancing, improved hygiene, and information seeking (22).

However, existing health anxiety scales and their cutoff scores were developed for general health concerns, not for the unique context of infectious disease outbreaks (e.g., anxiety about diseases like cancer; 23, 24). Infectious diseases present acute, population-wide threats that can rapidly shift what is considered adaptive versus maladaptive anxiety. As such, there is a critical need for measurement tools and empirically validated cutoffs specifically designed to assess health anxiety related to infectious diseases, to support research, clinical care, and public health responses.

To address this gap, the present study developed and validated the Peking Health Anxiety Scale for Infectious Diseases (PHAID). The scale adapts items from the widely used Short Health Anxiety Inventory (SHAI, 23), a decision grounded in its strong psychometric properties and its alignment with the cognitive-behavioral model of health anxiety. While developed in China, the PHAID was validated in an international sample to assess its utility beyond a single cultural context, acknowledging the global nature of infectious disease threats and as a first step toward establishing broader cross-cultural validity.

This study aimed to: (1) develop the PHAID and examine its factor structure; (2) evaluate its reliability, validity, and test-retest stability; (3) establish a preliminary clinical cutoff score; and (4) develop and validate a psychometrically sound short form (PHAID-S). We hypothesized that the PHAID would demonstrate a stable factor structure, excellent reliability, and strong validity, providing a context-sensitive tool to enhance the identification of clinically significant health anxiety during infectious disease outbreaks.

## 2 Methods

### 2.1 Participants

Data was collected at two points. At Time 1 (August-October 2020), 1,660 participants were recruited from Amazon Mechanical Turk (MTurk), an international platform accessible to anyone with internet and English proficiency. No geographical restrictions were applied, resulting in a naturalistic, international sample primarily from the United States (n = 1,136, 68.4%), India (n = 209, 12.6%), Brazil (n = 117, 7.0%), Canada (n = 66, 4.0%), Italy (n = 38, 2.3%), and 94 participants (5.6%) from 29 other countries. Participants were compensated $0.90 USD. The study was approved by the Peking University IRB (No.: 2020-04-18).

Eligibility criteria included informed consent, correct responses to an embedded validity check (judging the size of a common animal relative to a human; 25). Individuals with a confirmed COVID-19 diagnosis were excluded. This was necessary to ensure the scale specifically measured the fear of contracting the virus, rather than capturing the distinct psychological responses, such as illness-related distress or anxiety about recovery, that follow a confirmed infection, which would confound the construct of interest. The final Time 1 sample comprised 821 men, 830 women, and 9 individuals of other genders, with a mean age of 36.94 years (SD = 11.97).

Time 2 data were collected one month after Time 1, in November 2020. Following the same inclusion criteria, 355 participants were retained (181 men and 174 women), with a mean age of 39.21 years (SD = 12.69). Due to the high attrition rate (21.4%), a logistic regression analysis was conducted to examine potential attrition bias between participants who completed both time points (n = 355) and those who completed only the baseline assessment (n = 1,305). The analysis revealed that younger age (OR = 0.97, *p* < 0.001), higher baseline health anxiety (OR = 1.06, *p* < 0.001), and US residency (OR = 1.69, *p* < 0.001) were significant predictors of attrition. While this indicates some systematic patterns in dropout, the follow-up sample remains adequate for test-retest reliability analysis, though estimates should be interpreted with the understanding that they may be slightly conservative due to the selective attrition of more anxious participants.

The participant’s characteristics are shown in **Supplementary Table S1** in the supplementary material.

### 2.2 Measures

#### 2.2.1 Peking Health Anxiety Scale for Infectious Diseases

The PHAID is a 12-item scale designed to assess health anxiety in the context of infectious disease outbreaks, with its initial validation focused on COVID-19. The PHAID was adapted from the SHAI through a structured process to ensure content validity. First, an expert panel of two doctoral-level clinical psychologists systematically revised all items to focus on COVID-19-related health anxiety. Next, this adapted version was pilot-tested with a group of psychology students and practicing therapists to evaluate item clarity and relevance. Their feedback was used to refine the wording, resulting in the final item pool for psychometric evaluation. The measure was developed by adapting an initial pool of 18 items from the Short Health Anxiety Inventory (26). The content was revised to specify COVID-19 by (1) replacing general references to “illness” with “COVID-19,” (2) emphasizing the risk of contagion (e.g., “I am afraid of contracting COVID-19”). The reference period was set as “during the past six months,” corresponding to the pandemic timeframe. All items are rated on a 4-point Likert scale (1 = never to 4 = always), with higher scores indicating greater COVID-19-related health anxiety. After exploratory factor analysis, four items were removed due to low loadings or excessive cross-loading, yielding the final 12-item version. Although the PHAID was developed for COVID-19, its structure allows for straightforward adaptation to other public health emergencies. By substituting the relevant disease name in each item, the scale can be rapidly tailored to assess health anxiety related to future infectious disease outbreaks or similar public health events.

#### 2.2.2 Hospital Anxiety and Depression Scale

The HADS (27) is a 14-item scale with two 7-item subscales for anxiety (HADS-A) and depression (HADS-D). Each item is rated from 0 to 3, with higher scores indicating greater symptom severity. A subscale score of 8 or above indicates clinically significant anxiety or depression (28). Cronbach’s alpha in this study was.74.

#### 2.2.3 Handwashing

Handwashing was measured with four items, each assessing the frequency of proper hand hygiene (using soap and water or hand sanitizer) in a specific scenario during the past three days: arriving home, before cooking or eating, after coughing or sneezing, and after using the restroom. All items are rated on a 5-point Likert scale (1 = never to 5 = always). Cronbach’s alpha in this study was.81.

### 2.3 Statistical analysis

All analyses were conducted using SPSS 25, Mplus 8.3, and R. Parallel analysis, exploratory factor analysis (EFA), assessments of convergent and discriminant validity, internal consistency, test-retest reliability, Receiver Operating Characteristic (ROC) analysis, and Pearson correlations were performed in SPSS. Confirmatory factor analysis (CFA) was conducted in Mplus. Item Response Theory (IRT) analysis was performed in R using the *ltm* package.

To determine the factor structure of the PHAID, the full Time 1 sample (n = 1,660) was randomly split into two equal subsamples (n = 830 each). The first subsample was used for EFA. Prior to EFA, a parallel analysis was conducted to determine the number of factors to retain, which involved comparing actual data eigenvalues with the 95th percentile of eigenvalues from 1,000 simulated random datasets (29, 30). Factors were retained if the *i*-th eigenvalue from the data exceeded the corresponding random value (31, 32). EFA was performed using principal axis factoring with Promax (oblique) rotation (33). Items were iteratively removed if they had a factor loading below 0.35 or a cross-loading difference less than 0.2 between primary and secondary factors.

A series of CFAs were conducted in the second subsample to evaluate and compare three competing models: (1) the original SHAI factor structure, (2) the two-factor structure derived from EFA, and (3) a modified version of the EFA-based model. For the EFA-derived model, items with loadings below 0.50 were excluded. In line with methodological recommendations to avoid overfitting (34), model modifications were made only for a limited number of item pairs that demonstrated both high modification indices (MI > 10, 35) and a strong, *a priori* substantive rationale based on close semantic content. The model fit was evaluated against conventional standards (36). An adequate model fit was indicated by 1 < *χ*^2^/*df* < 3, comparative fit index (CFI) and Tucker Lewis index (TLI) values ≥ 0.90, root-mean-square-error of approximation (RMSEA) ≤ 0.08 (more liberal) or ≤ 0.05 (stricter), and standardized root-mean-square residual (SRMR) value ≤ 0.05.

Scale validity and reliability were assessed via multiple methods. Convergent validity was evaluated by correlating the PHAID total score with the HADS anxiety subscale; discriminant validity was assessed via correlation with the HADS depression subscale. Internal consistency was estimated using Cronbach’s alpha for the total scale and subscales. Test-retest reliability was assessed by correlating PHAID scores at Time 1 and at the one-month follow-up (Time 2).

To establish a clinical cutoff for the PHAID, ROC analysis was conducted using the HADS anxiety subscale and its established cutoff (28) as the reference standard. Regression analyses were performed to examine the relationship between PHAID total score and hand washing total score. Multiple models (linear, quadratic, cubic, power, logarithmic, and exponential) were tested, and model fit was compared using adjusted R². Notably, the regression analysis examining the relationship between PHAID scores and handwashing was conducted for exploratory purposes to investigate the form of this relationship, rather than to build a predictive model.

Item Response Theory (IRT) analysis was conducted to inform the development of a short form (PHAID-S). A Graded Response Model (GRM, 37) was fitted to the data. For each item, two parameters were estimated: a discrimination parameter (a) and three difficulty parameters (b1, b2, b3), corresponding to the thresholds between the four ordered response categories (0-3). Test Information Curves (TIC) and Item Information Curves (IIC) were also calculated to evaluate the measurement precision of the scale and its individual items across the latent trait of health anxiety. A ROC analysis was also conducted to establish a cutoff of PHAID-S.

## 3 Results

### 3.1 Exploratory factor analysis

The data were suitable for EFA, as indicated by a significant Bartlett’s test of sphericity (*p* <.001) and a high Kaiser-Meyer-Olkin (KMO) measure of sampling adequacy (0.945). Parallel analysis supported the extraction of two factors, with the actual eigenvalues for the first two factors exceeding the 95th percentile eigenvalues from simulated random data (see **Supplementary Table S2** in supplementary material). Item 4 was removed due to a factor loading below 0.30, and Items 17, 15, and 3 were subsequently removed due to excessive cross-loadings. The final solution, presented in **Table 1**, consisted of two factors that collectively accounted for 61.93% of the total variance. All retained items demonstrated strong loadings, ranging from 0.51 to 0.89. Negative cross-loadings are a known artifact of oblique rotation and should be interpreted in the context of the strong primary loadings and the inter-factor correlation. Based on the content of the items, Factor 1 was labeled *Catastrophic Thinking*, and Factor 2 was labeled *Infection Worries*.

**Table 1:** Result of exploratory factor analysis (EFA).

Items	Factors 1 catastrophic thinking	Factors 2 infection worries
9. If I hear about COVID-19, I think I have it myself.	**0.886**	-0.203
12. I think that I have COVID-19.	**0.880**	-0.088
18. If I had COVID-19, I would feel that I had lost my dignity.	**0.841**	-0.129
13. If I notice an unexplained bodily sensation, I would think about COVID-19 all the time, and I find it difficult to think about other things.	**0.807**	0.032
7. I have difficulty taking my mind off thoughts about COVID-19.	**0.781**	0.113
14. My family/friends would say I worry too much about COVID-19.	**0.769**	0.069
6. I have images of myself getting COVID-19.	**0.739**	0.111
10. If I have a bodily sensation or change, I wonder whether I have COVID-19.	**0.737**	0.142
11. I usually feel at risk for developing COVID-19.	**0.629**	0.259
16. If I have COVID-19, there is a small chance that modern medicine would be able to cure me.	**0.510**	0.045
1. I worry about contracting COVID-19	0.113	**0.809**
5. I am afraid of contracting COVID-19.	0.219	**0.749**
2. I am more afraid of contracting COVID-19 compared with most other people of my age.	-0.125	**0.603**
8. I am relieved if my doctor tells me I do not have COVID-19.	0.378	**0.593**

Bolded numbers indicate the highest factor loading for each item on its respective factor.

### 3.2 Confirmatory factor analysis

**Figure 1:**
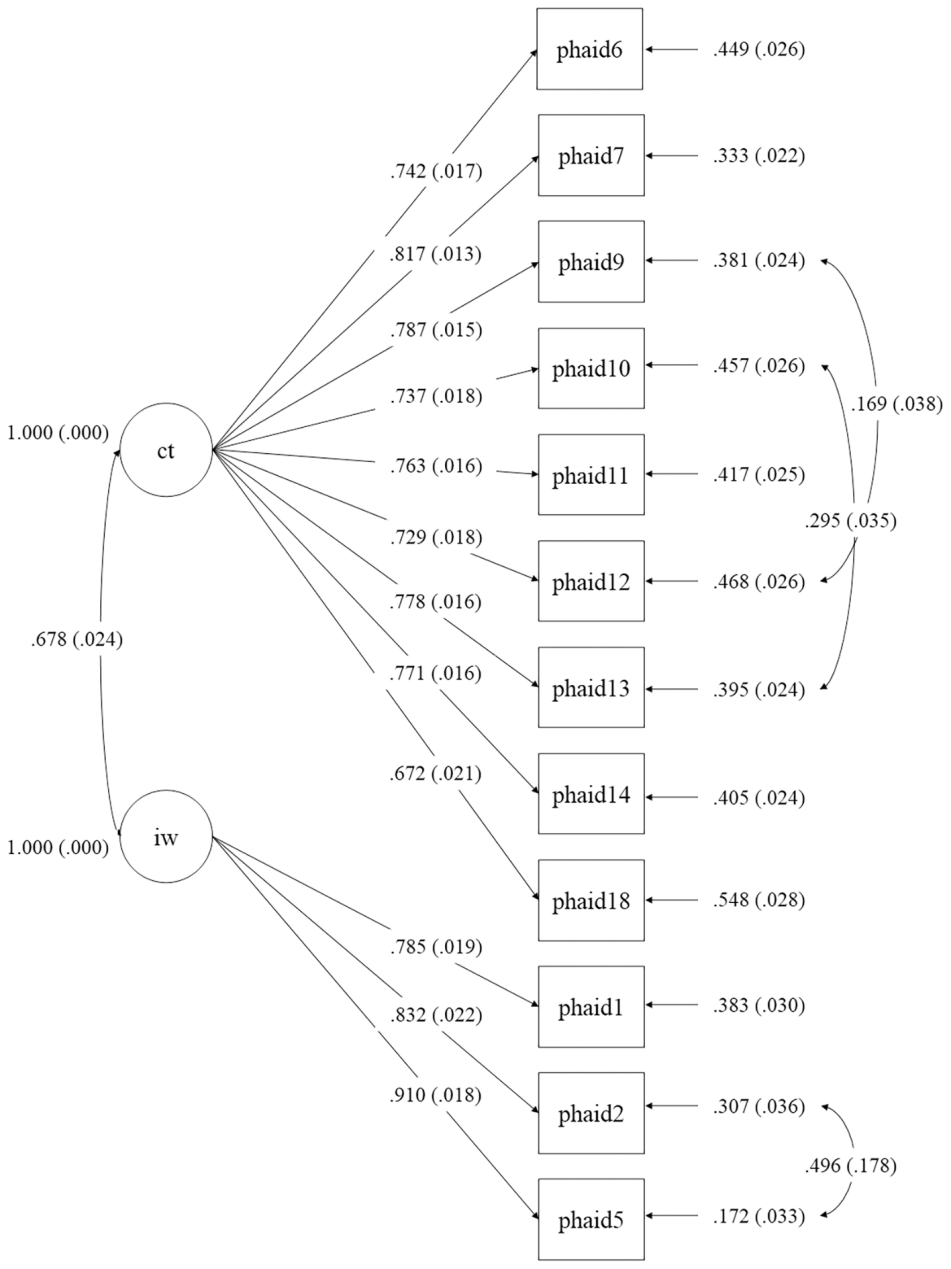
Path diagram of the revised two-factor structure from confirmatory factor analysis (CFA) of the PHAID, showing standardized factor loadings. ct, Catastrophic Thinking; iw, Infection Worries. Standardized coefficients were shown.

The original SHAI factor structure showed all standardized factor loadings were statistically significant (*p* <.01 for item 4; *p* <.001 for all others); however, model fit was poor: χ²/df = 10.04, *p* <.001, CFI = .843, TLI = .821, RMSEA = .104, SRMR = .061. The two-factor EFA-derived structure required the removal of Items 8 and 16 due to loadings below 0.50. All remaining items loaded significantly (*p* <.001), and model fit improved substantially: χ²/df = 7.94, *p* <.001, CFI = .941, TLI = .927, RMSEA = .091, SRMR = .046. Moreover, error covariances were added for three pairs: item 12 and 9 (self-diagnosis), item 13 and 10 (misinterpretation of bodily sensations), and item 5 and 2 (core fear of infection). The modified model (see **Figure 1**) demonstrated excellent fit: χ²/df = 6.53, *p* <.001, CFI = .956, TLI = .942, RMSEA = .082, SRMR = .040.

### 3.3 Internal consistency

As presented in **Table 2**, Pearson correlation analyses revealed strong and statistically significant positive associations (all *p* <.001). The correlations between the total score and the two subscales were very high, ranging from 0.619 to 0.975. Similarly, all items showed strong correlations with the total score (*r* = 0.634 to 0.821), and with their respective subscales (*r* = 0.756 to 0.835 for *Catastrophic Thinking*; *r* = 0.874 to 0.888 for *Infection Worries*). The Cronbach’s alpha and McDonald’s omega were excellent: *α* = 0.931 and ω = .947 for the total scale, *α* = 0.928 and ω = .939 for the *Catastrophic Thinking* subscale, and *α* = 0.851 and ω = .856 for the *Infection Worries* subscale.

**Table 2:** Pearson correlations of PHAID total and subscale scores with each item.

Dimensions/items	Total score	Catastrophic thinking	Infection worries
Catastrophic Thinking	0.975***	–	–
Infection Worries	0.777***	0.619***	–
1. I worry about contracting COVID-19.	0.634***	–	0.876***
2. I am more afraid of contracting COVID-19 compared with most other people of my age.	0.719***	–	0.874***
5. I am afraid of contracting COVID-19.	0.692***	–	0.888***
6. I have images of myself getting COVID-19.	0.780***	0.789***	–
7. I have difficulty taking my mind off thoughts about COVID-19.	0.821***	0.826***	–
9. If I hear about COVID-19, I think I have it myself.	0.774***	0.793***	–
10. If I have a bodily sensation or change, I wonder whether I have COVID-19.	0.785***	0.766***	–
11. I usually feel at risk for developing COVID-19.	0.736***	0.786***	–
12. I think that I have COVID-19.	0.797***	0.821***	–
13. If I notice an unexplained bodily sensation, I would think about COVID-19 all the time, and I find it difficult to think about other things.	0.716***	0.756***	–
14. My family/friends would say I worry too much about COVID-19.	0.634***	0.789***	–
18. If I had COVID-19, I would feel that I had lost my dignity.	0.692***	0.835***	–

***p <.001.

### 3.4 Test–retest reliability

One-month test–retest correlations were high for the total score (*r* = 0.826, *p* <.001), as well as for the *Catastrophic Thinking* (*r* = 0.780, *p* <.001) and *Infection Worries* (*r* = 0.821, *p* <.001) subscales.

### 3.5 Convergent and discriminant validity

The PHAID total score and its subscales showed significant, moderate-to-strong positive correlations with the HADS anxiety subscale, supporting convergent validity (PHAID total: *r* = 0.622, *Catastrophic Thinking*: *r* = 0.617, *Infection Worries*: *r* = 0.454; all *ps* <.01). In contrast, correlations with the HADS depression subscale were weaker (PHAID total: *r* = 0.263, *Catastrophic Thinking*: *r* = 0.267, *Infection Worries*: *r* = 0.175; all *ps* <.01). The association between the PHAID total score and anxiety was significantly greater than with depression (*Z****_H_*** = 15.45, *p* <.001), supporting the convergent and discriminant validity of the PHAID.

### 3.6 ROC analysis for cutoff determination

**Figure 2:**
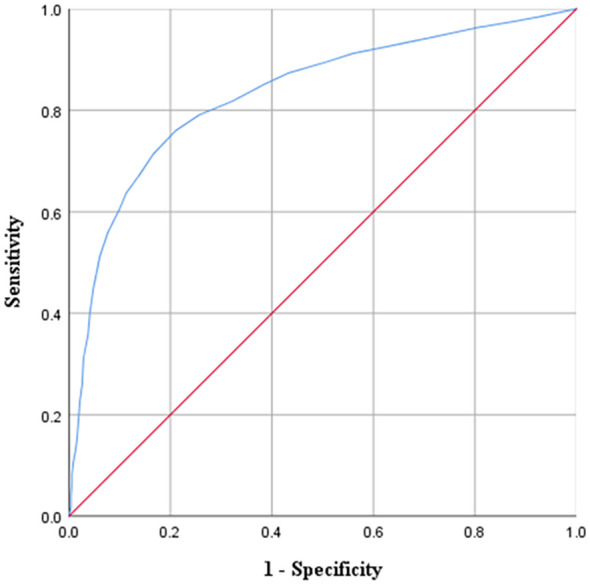
ROC curves for PHAID. PHAID, Peking Health Anxiety Scale for Infectious Diseases.

The ROC curve for the PHAID, using the HADS anxiety subscale (cutoff ≥ 8) as the criterion, yielded an area under the curve (AUC) of 0.83 (*p* <.001, 95% CI [0.81, 0.85]; see **Figure 2**). The optimal cutoff score was 24 on the PHAID total, corresponding to the maximum Youden’s index of 0.550. At this threshold, sensitivity was 0.760 and specificity was 0.790. Sensitivity, specificity, and Youden’s index values for additional cutoff scores are presented in **Supplementary Table S3** in supplementary material.

**Figure 3:**
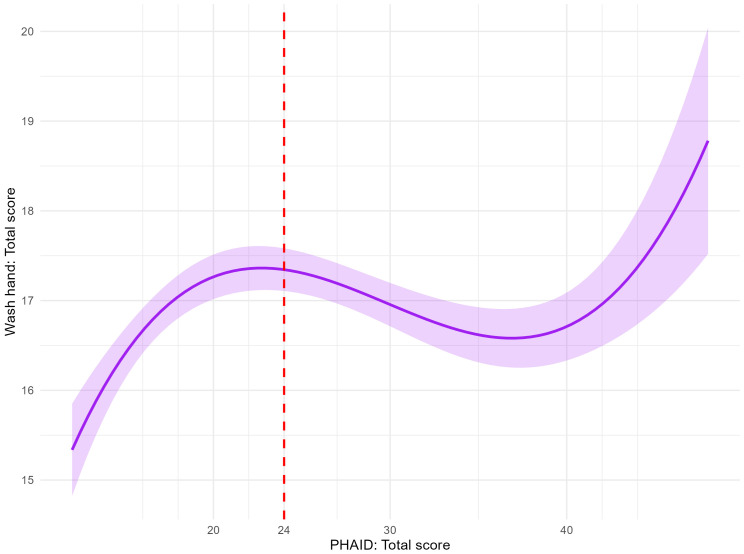
Cubic regression of PHAID scores on hand washing total score. PHAID, Peking Health Anxiety Scale for Infectious Disease. The red dashed line indicates the PHAID cutoff point of 24.

The relationship between PHAID total scores and hand washing total score was best described by a cubic regression model (adjusted R² = .02), as shown in **Figure 3**. The cubic model demonstrated a significant overall fit, F(3, 1656) = 13.81, *p* <.001. All predictors were statistically significant: linear term (b = 1.39, t = 5.91, *p* <.001), quadratic term (b = −0.05, t = −5.60, *p* <.001), and cubic term (b = 0.0006, t = 5.30, *p* <.001). Compared to the cubic model, the quadratic, power, logarithmic, exponential, and linear models accounted for less variance (adjusted R² = .007,.005,.005,.002, and.002, respectively). The cubic regression curve indicated that hand washing total score increased with PHAID scores up to a total score of 24, plateaued between scores of 24 and 36, and increased again at higher scores. It is important to note that this regression analysis was exploratory in nature. The low adjusted R² confirms that health anxiety is just one of many factors influencing handwashing, and the PHAID is not intended to predict this behavior. The key finding is the significant non-linear pattern itself, which provides preliminary construct validity by aligning with the theoretical model of adaptive versus maladaptive anxiety.

### 3.7 Item Response Theory analysis for short-form development

All items showed discrimination parameters greater than 2.87. For *Catastrophic Thinking*, Items 9 (a = 3.21, b1 = -0.08, b2 = 0.74, b3 = 1.63), 13 (a = 2.89, b1 = -0.39, b2 = 0.66, b3 = 1.72), and 7 (a = 2.87, b1 = -0.43, b2 = 0.65, b3 = 1.63) had the highest discrimination and provided the most information according to the Item Information Curves. For *Infection Worry*, Items 5 (a = 3.68, b1 = -1.39, b2 = -0.03, b3 = 0.98) and 1 (a = 3.38, b1 = -1.67, b2 = -0.16, b3 = 0.96) had the highest discrimination and information values. These five items were selected for the PHAID-S. The 5 selected items adequately represent the core content of the original factors: the *Catastrophic Thinking* factor is represented by three high-discrimination items covering difficulty disengaging from thoughts, self-diagnosis, and persistent rumination, while the *Infection Worries* factor is represented by its two most central items (general worry and fear).

**Figure 4:**
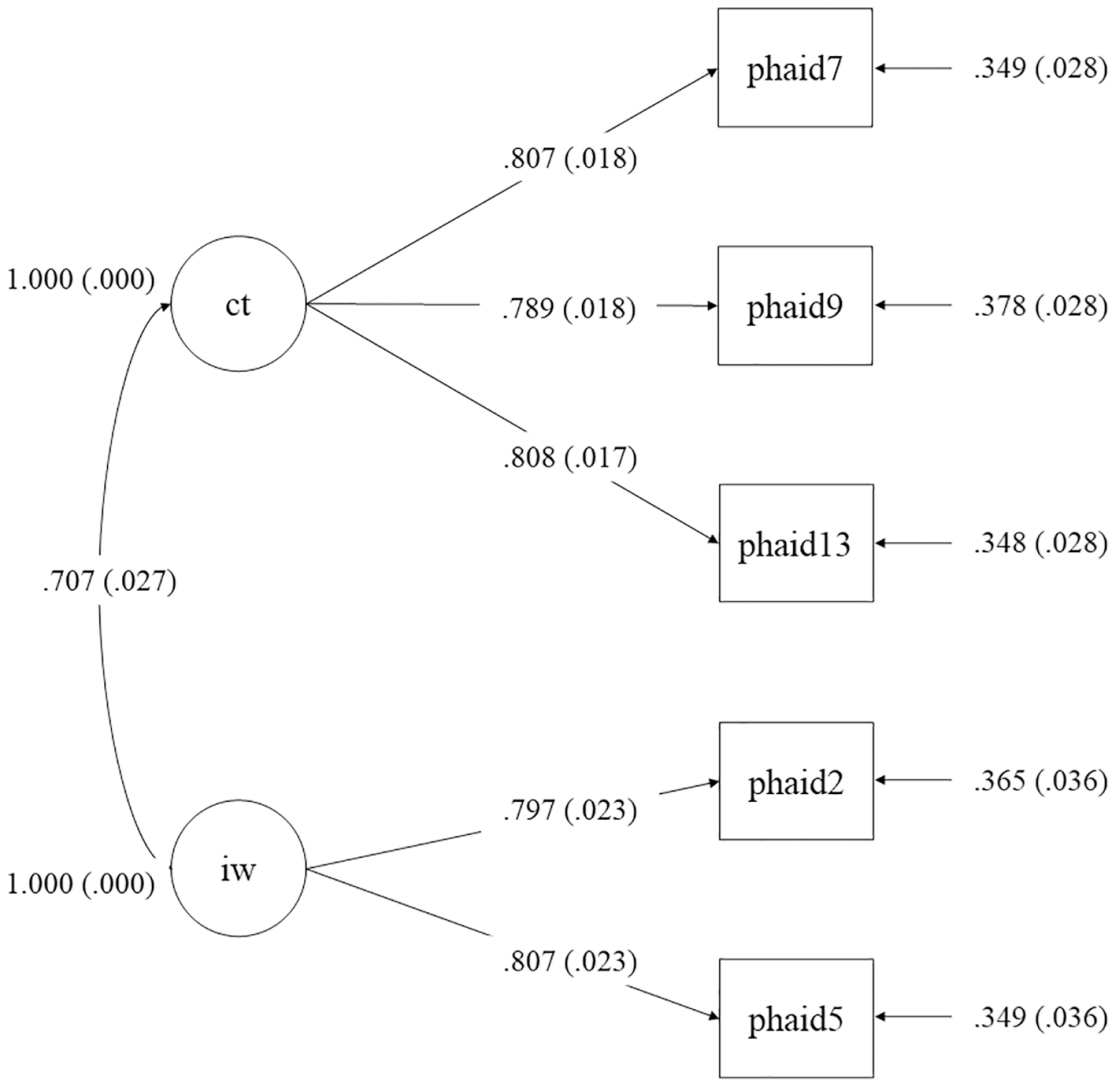
Path diagram of the two-factor structure from confirmatory factor analysis (CFA) of the PHAID-S, showing standardized factor loadings. ct, Catastrophic Thinking; iw, Infection Worries. Standardized coefficients were shown.

Confirmatory factor analysis was conducted to evaluate the two-factor structure of the 5-item PHAID-S. The model demonstrated an excellent fit to the data: χ²/df = 9.55, *p* <.001, CFI = .981, TLI = .953, RMSEA = .102, SRMR = .026. All standardized factor loadings were statistically significant (*p* <.001) and substantial, ranging from 0.79 to 0.81 for the *Catastrophic Thinking* factor (Items 7, 9, 13) and from 0.80 to 0.81 for the *Infection Worries* factor (Items 2, 5), as shown in **Figure 4**.

Test Information Curves showed maximum measurement precision for *Catastrophic Thinking* in the latent trait range of -0.5 to 1.5, and for *Infection Worry* in the range of -1.5 to 1.0 for both PHAID and PHAID-S, indicating that the PHAID-S covers a similar range of the latent trait as the full PHAID, although with a lower peak information. The short form demonstrated internal consistency of Cronbach’s *α* = 0.85. Detailed parameter estimates and information curves are presented in **Supplementary Table S4** and **Supplementary Figures S1–S4** in the supplementary materials.

For the PHAID-S, ROC analysis (criterion: HADS anxiety subscale ≥ 8) yielded an AUC of 0.81 (*p* <.001, 95% CI [0.81, 0.85]; see **Supplementary Figure S3**). The optimal cutoff score for the PHAID-S was 11, corresponding to the maximum Youden’s index of 0.470. However, considering the utility of a short form for efficient screening, a cutoff of 12 may also be considered, as it offers substantially higher specificity (0.845 vs. 0.744) for a moderate decrease in sensitivity (0.653 vs. 0.727), which could be preferable in settings where minimizing false positives is a priority. The cutoff of 11 is recommended for maximum sensitivity. Additional cutoff statistics are provided in **Supplementary Table S5** in the supplementary material.

## 4 Discussion

This study developed and validated the Peking Health Anxiety Scale for Infectious Diseases (PHAID), a domain-specific screener designed to identify clinically significant anxiety related to transmissible illnesses. Through psychometric evaluation in a large sample primarily recruited via an online platform MTurk (*n* = 1,660), we established a stable two-factor structure (*Catastrophic Thinking* and *Infection Worries*) with excellent reliability (total scale α = 0.931, test-retest *r* = 0.826) and validity (stronger correlation with anxiety than depression). Although developed during the COVID-19 pandemic, the PHAID’s focus on common respiratory symptoms (e.g., cough, fever, dyspnea) allows for its adaptation to similar infectious disease contexts, including H1N1 influenza and SARS (38, 39). The PHAID offers a practical framework for assessing infection-focused cognitions and worries during public health emergencies.

The PHAID is the first measure specifically adapted and validated during the COVID-19 pandemic to assess the distinct construct of health anxiety within an infectious disease context. The research landscape has seen the rapid development of various tools to capture pandemic-related distress, including scales for fear (fear of COVID-19 scale, FCV-19S; 40), unidimensional anxiety (COVID-19 anxiety scale, CAS; 41, 42), maladaptive coping syndromes (COVID-19 anxiety syndrome scale, C-19ASS; 43), and broader stress responses (COVID Stress Scales, CSS; 44). (a) lack specificity for its defining cognitive features (e.g., the FCV-19S); (b) focus on the behavioral manifestations and coping strategies rather than the core health-related fears (e.g., the C-19ASS); (c) are often impractical for rapid screening due to their length and complexity (e.g., the CSS); and crucially, (d) are not theoretically grounded in the cognitive-behavioral model of health anxiety. The PHAID directly addresses these gaps by providing a concise, theoretically grounded tool that specifically targets and dissects the core cognitions (Catastrophic Thinking) and health-related fears (Infection Worries) characteristic of infectious disease health anxiety, thereby enabling precise identification and intervention (45).

The screening utility and clinical relevance of the PHAID were strengthened by establishing an empirically derived cutoff score of 24. ROC analysis demonstrated a favorable balance between sensitivity (76%) and specificity (79%), with performance characteristics comparable to established psychiatric screening tools, such as the State-Trait Inventory for Cognitive and Somatic Anxiety (STICSA; 46) and the General Health Questionnaire (GHQ; 47). The clinical significance of this cutoff was supported through behavioral validation examining the relationship between PHAID scores and handwashing frequency. An exploratory cubic regression analysis revealed a significant, theoretically meaningful pattern: scores approaching the cutoff were associated with increasing hand hygiene, consistent with adaptive health vigilance (22). Beyond this threshold, protective behaviors plateaued, while severely elevated scores (around 36) showed renewed increases—a pattern potentially indicating a shift toward compulsive responding (48). Although this preliminary finding requires further validation, it suggests the PHAID may help differentiate adaptive vigilance from potentially excessive anxiety responses, supporting its construct validity as a tool to identify individuals who could benefit from further assessment during outbreaks.

The study also developed a 5-item short form (PHAID-S) that maintains good reliability (α = 0.85) despite substantial length reduction. Derived through Item Response Theory, the PHAID-S retains high discrimination power in the low-to-moderate severity range, making it well-suited for rapid screening in time-sensitive clinical settings, large-scale public health surveys, and repeated monitoring during treatment. This brief format provides an efficient alternative for initial screening, while the full scale remains preferable for comprehensive assessment requiring finer gradations, particularly at higher severity levels.

### 4.1 Limitations & future directions

Several limitations of this study should be acknowledged. First, participants were recruited via Amazon Mechanical Turk (MTurk). While this platform enabled the collection of a geographically diverse sample, the participant pool may overrepresent. White, middle-class individuals with higher education levels and computer literacy. Moreover, while our sample included participants from multiple countries, its online nature and Western predominance warrant caution regarding cultural generalizability. Second, the data were collected during the early stages of the COVID-19 pandemic (August-November 2020). The psychological impact and associated health anxiety may have evolved as the pandemic progressed, and public health policies varied across regions. Consequently, the results might underestimate the potential severity of such anxiety in later phases of the pandemic or in regions experiencing severe outbreaks. Third, the test-retest reliability assessment was limited by the sample size, and attrition analysis revealed that the dropout was not completely random but was influenced by age, nationality, and baseline health anxiety scores. Future studies should collect more unbiased longitudinal data to further examine the scale’s temporal stability. Fourth, while the PHAID demonstrates good convergent validity with general anxiety (HADS-A) and discriminant validity from depression (HADS-D), its direct convergent validity with a measure of health anxiety itself (e.g., the full Short Health Anxiety Inventory) was not assessed in this study. Fifth, the interpretation of the significant cubic relationship with handwashing is exploratory and requires validation in studies specifically designed to test this behavioral pattern.

Future research can focus on several key areas to extend the utility of the PHAID. First, validating the PHAID and its short form across more diverse populations—including varied cultural, socioeconomic, and clinical groups—would help establish its broader applicability and stability across different contexts. Second, future studies could explore how factors such as media exposure, health literacy, and pre-existing vulnerabilities influence PHAID. Third, future studies could investigate how varying levels of PHAID scores correlate with a spectrum of health behaviors, from appropriate vigilance to excessive actions. Fourth, the clinical and public health utility of the PHAID cutoff scores could be evaluated in real-world settings to assess their effectiveness in identifying individuals who might benefit from targeted support.

## 5 Conclusion

The PHAID demonstrates good psychometric properties through its validated two-factor structure, excellent reliability, and empirically derived cutoff score, addressing a critical measurement gap in infectious disease-related health anxiety. As an early-stage tool, the PHAID shows potential to provide researchers and clinicians with an evidence-based instrument for identifying individuals who may benefit from targeted support during public health emergencies.

## Data Availability

The raw data supporting the conclusions of this article will be made available by the authors, without undue reservation.

## References

[1] Abramowitz JS Braddock A . Psychological treatment of health anxiety and hypochondriasis: A biopsychosocial approach. Göttingen, Germany: Hogrefe publishing (2008).

[2] Bailer J Kerstner T Witthoft M Diener C Mier D Rist F . Health anxiety and hypochondriasis in the light of DSM-5. Anxiety Stress Coping. (2016) 29:219–39. doi: 10.1080/10615806.2015.1036243, PMID: 25846805

[3] Jungmann SM Witthoft M . Health anxiety, cyberchondria, and coping in the current COVID-19 pandemic: Which factors are related to coronavirus anxiety? J Anxiety Disord. (2020) 73:102239. doi: 10.1016/j.janxdis.2020.102239, PMID: 32502806 PMC7239023

[4] Taylor S Asmundson GJ . Treating health anxiety: A cognitive-behavioral approach. New York, USA: Guilford Press New York (2004).

[5] Marcus DK Gurley JR Marchi MM Bauer C . Cognitive and perceptual variables in hypochondriasis and health anxiety: a systematic review. Clin Psychol Rev. (2007) 27:127–39. doi: 10.1016/j.cpr.2006.09.003, PMID: 17084495

[6] Salkovskis PM Warwick HM . Morbid preoccupations, health anxiety and reassurance: a cognitive-behavioural approach to hypochondriasis. Behav Res Ther. (1986) 24:597–602. doi: 10.1016/0005-7967(86)90041-0, PMID: 3753387

[7] Stenning NJ . A meta-analytic investigation of the psychometric properties of the Health Anxiety Questionnaire and the Health Anxiety Inventory University of Leeds. (2017).

[8] Liu B Yang X Kong L . Classification of the infectious diseases. In: Li H , editor. Radiology of infectious diseases: volume 1. Philadelphia, USA: Springer Netherlands (2015). p. 29–30. doi: 10.1007/978-94-017-9882-2_5

[9] Bai Y Yao LS Wei T Tian F Jin DY Chen LJ . Presumed asymptomatic carrier transmission of COVID-19. Jama-Journal Am Med Assoc. (2020) 323:1406–7. doi: 10.1001/jama.2020.2565, PMID: 32083643 PMC7042844

[10] Dennis D Radnitz C Wheaton MG . A perfect storm? Health anxiety, contamination fears, and COVID-19: lessons learned from past pandemics and current challenges. Int J Cognit Ther. (2021) 14:351–67. doi: 10.1007/s41811-021-00109-7, PMID: 33907592 PMC8061445

[11] Huang CL Wang YM Li XW Ren LL Zhao JP Hu Y . Clinical features of patients infected with 2019 novel coronavirus in Wuhan, China. Lancet. (2020) 395:497–506. doi: 10.1016/S0140-6736(20)30183-5, PMID: 31986264 PMC7159299

[12] Lu JY Yang ZC . COVID-19 outbreak associated with air conditioning in restaurant, guangzhou, China 2020 Response. Emerging Infect Dis. (2020) 26:2790–2. doi: 10.3201/eid2611.203774, PMID: 32917292 PMC7588507

[13] Rasmussen AL Popescu SV . SARS-CoV-2 transmission without symptoms. Science. (2021) 371:1206–7. doi: 10.1126/science.abf9569, PMID: 33737476

[14] Zhang HN Li X Ma RH Li XX Zhou YF Dong HL . Airborne spread and infection of a novel swine-origin influenza A (H1N1) virus. Virol J. (2013) 10:204. doi: 10.1186/1743-422x-10-204, PMID: 23800032 PMC3700749

[15] Kibbey MM Fedorenko EJ Farris SG . Anxiety, depression, and health anxiety in undergraduate students living in initial US outbreak “hotspot” during COVID-19 pandemic. Cogn Behav Ther. (2021) 50:409–21. doi: 10.1080/16506073.2020.1853805, PMID: 33433271

[16] Li MY Liu L Yang YL Wang Y Yang XS Wu H . Psychological impact of health risk communication and social media on college students during the COVID-19 pandemic: cross-sectional study. J Med Internet Res. (2020) 22:e20656. doi: 10.2196/20656, PMID: 33108308 PMC7677589

[17] Elhai JD McKay D Yang HB Minaya C Montag C Asmundson GJG . Health anxiety related to problematic smartphone use and gaming disorder severity during COVID-19: Fear of missing out as a mediator. Hum Behav Emerging Technol. (2021) 3:137–46. doi: 10.1002/hbe2.227, PMID: 33363275 PMC7753448

[18] Tyrer P . Recent advances in the understanding and treatment of health anxiety. Curr Psychiatry Rep. (2018) 20:49. doi: 10.1007/s11920-018-0912-0, PMID: 29931576

[19] Garboczy S Szeman-Nagy A Ahmad MS Harsanyi S Ocsenas D Rekenyi V . Health anxiety, perceived stress, and coping styles in the shadow of the COVID-19. BMC Psychol. (2021) 9:53. doi: 10.1186/s40359-021-00560-3, PMID: 33823945 PMC8022303

[20] Jokic-Begic N Korajlija AL Mikac U . Cyberchondria in the age of COVID-19. PloS One. (2020) 15:e0243704. doi: 10.1371/journal.pone.0243704, PMID: 33332400 PMC7746178

[21] Trougakos JP Chawla N McCarthy JM . Working in a pandemic: exploring the impact of COVID-19 health anxiety on work, family, and health outcomes. J Appl Psychol. (2020) 105:1234–45. doi: 10.1037/apl0000739, PMID: 32969707

[22] Marschalko EE Kotta I Kalcza-Janosi K Szabo K Jancso-Farcas S . Psychological predictors of COVID-19 prevention behavior in hungarian women across different generations. Front Psychol. (2021) 12:596543. doi: 10.3389/fpsyg.2021.596543, PMID: 33574787 PMC7870484

[23] Alberts NM Hadjistavropoulos HD Jones SL Sharpe D . The Short Health Anxiety Inventory: A systematic review and meta-analysis. J Anxiety Disord. (2013) 27:68–78. doi: 10.1016/j.janxdis.2012.10.009, PMID: 23247202

[24] Hedman E Lekander M Ljotsson B Lindefors N Ruck C Andersson G . Optimal cut-off points on the health anxiety inventory, illness attitude scales and whiteley index to identify severe health anxiety. PloS One. (2015) 10:e0123412. doi: 10.1371/journal.pone.0123412, PMID: 25849477 PMC4388630

[25] Barger P Behrend T Sharek D Sinar E . IO and the crowd: Frequently asked questions about using Mechanical Turk for research. Industrial-Organizational Psychol. (2011) 49:11–7.

[26] Salkovskis PM Rimes KA Warwick HMC Clark DM . The Health Anxiety Inventory: development and validation of scales for the measurement of health anxiety and hypochondriasis. psychol Med. (2002) 32:843–53. doi: 10.1017/S0033291702005822, PMID: 12171378

[27] Zigmond AS Snaith RP . The hospital anxiety and depression scale. Acta Psychiatr Scand. (1983) 67:361–70. doi: 10.1111/j.1600-0447.1983.tb09716.x, PMID: 6880820

[28] Bjelland I Dahl AA Haug TT Neckelmann D . The validity of the Hospital Anxiety and Depression Scale. An updated literature review. J Psychosom Res. (2002) 52:69–77. doi: 10.1016/s0022-3999(01)00296-3, PMID: 11832252

[29] Hayton JC Allen DG Scarpello V . Factor retention decisions in exploratory factor analysis: A tutorial on parallel analysis. Organizational Res Methods. (2004) 7:191–205. doi: 10.1177/1094428104263675

[30] O’Connor BP . SPSS and SAS programs for determining the number of components using parallel analysis and Velicer’s MAP test. Behav Res Methods Instruments Comput. (2000) 32:396–402. doi: 10.3758/Bf03200807, PMID: 11029811

[31] Ford JK Maccallum RC Tait M . The application of exploratory factor-analysis in applied-psychology - a critical-review and analysis. Personnel Psychol. (1986) 39:291–314. doi: 10.1111/j.1744-6570.1986.tb00583.x

[32] Lautenschlager GJ . A comparison of alternatives to conducting monte-carlo analyses for determining parallel analysis criteria. Multivariate Behav Res. (1989) 24:365–95. doi: 10.1207/s15327906mbr2403_6, PMID: 26750503

[33] de Winter JCF Dodou D . Factor recovery by principal axis factoring and maximum likelihood factor analysis as a function of factor pattern and sample size. J Appl Stat. (2012) 39:695–710. doi: 10.1080/02664763.2011.610445

[34] Hermida R . The problem of allowing correlated errors in structural equation modeling: concerns and considerations. Comput Methods Soc Sci. (2015) 3:5–17.

[35] Lomax RG . A beginner’s guide to structural equation modeling. New York, USA: Psychology press (2004).

[36] Hu LT Bentler PM . Cutoff criteria for fit indexes in covariance structure analysis: conventional criteria versus new alternatives. Struct Equation Modeling-a Multidiscip J. (1999) 6:1–55. doi: 10.1080/10705519909540118

[37] Rizopoulos D . ltm: An R package for latent variable modeling and item response analysis. J Stat Software. (2007) 17:1–25.

[38] Lee CS Lee JH . Dynamics of clinical symptoms in patients with pandemic influenza A (H1N1). Clin Microbiol Infect. (2010) 16:389–90. doi: 10.1111/j.1469-0691.2010.03117.x, PMID: 20222893

[39] Peiris JS Guan Y Yuen KY . Severe acute respiratory syndrome. Nat Med. (2004) 10:S88–97. doi: 10.1038/nm1143, PMID: 15577937 PMC7096017

[40] Ahorsu DK Lin C-Y Imani V Saffari M Griffiths MD Pakpour AH . The fear of COVID-19 scale: development and initial validation. Int J Ment Health Addict. (2022) 20:1537–45. doi: 10.1007/s11469-020-00270-8, PMID: 32226353 PMC7100496

[41] Lee SA . Coronavirus Anxiety Scale: A brief mental health screener for COVID-19 related anxiety. Death Studies (2020) 44:393–401. doi: 10.1080/07481187.2020.1748481, PMID: 32299304

[42] Silva WAD de Sampaio Brito TR Pereira CR. COVID-19 anxiety scale (CAS): Development and psychometric properties. Curr Psychol (2022) 41:5693–702. doi: 10.1007/s12144-020-01195-0, PMID: 33204058 PMC7661558

[43] Nikčević AV Spada MM . The COVID-19 anxiety syndrome scale: Development and psychometric properties. Psychiatry research (2020) 292:113322. doi: 10.1016/j.psychres.2020.113322, PMID: 32736267 PMC7375349

[44] Taylor S Landry CA Paluszek MM Fergus TA McKay D Asmundson GJ . Development and initial validation of the COVID Stress Scales. J Anxiety Disord. (2020) 72:102232. doi: 10.1016/j.janxdis.2020.102232, PMID: 32408047 PMC7198206

[45] Du X Lai L Shi C Guo Z Han J Zhang T . Internet-based cognitive bias modification of interpretation in health anxiety: A randomized controlled trial. Acta Psychologica Sin. (2024) 56:1351. doi: 10.3724/SP.J.1041.2024.01351

[46] Van Dam NT Gros DF Earleywine M Antony MM . Establishing a trait anxiety threshold that signals likelihood of anxiety disorders. Anxiety stress coping. (2013) 26:70–86. doi: 10.1080/10615806.2011.631525, PMID: 22091946

[47] Weinstein MC Berwick DM Goldman PA Murphy JM Barsky AJ . A comparison of three psychiatric screening tests using receiver operating characteristic (ROC) analysis. Med Care. (1989) 27:593–607. doi: 10.1097/00005650-198906000-00003, PMID: 2725088

[48] Johnsen L Bream V French S Morriss R Salkovskis PM . Evaluating CBT for health anxiety and obsessive compulsive disorder adapted for online delivery in the context of COVID-19. Behav Cogn Psychother (2025) 53(2):114–26. doi: 10.1017/S1352465824000511, PMID: 40065556

