# Supplementary material for: The Peking Health Anxiety Scale for Infectious Diseases: psychometric properties and short-form development
Source: Front Psychiatry. 2026 Jan 5;16:1734657. doi: 10.3389/fpsyt.2025.1734657 (PMC12812606; doi:10.3389/fpsyt.2025.1734657)

**Appendix S1: Influence of demographic variables on PHAID scores**

To examine the influence of demographic variables on PHAID scores, we conducted multiple regression analyses. For the PHAID total score, demographic factors (age, gender, country) collectively accounted for only 0.46% of the variance (R² = 0.0046, F(3, 1655) = 2.55, *p* = .054). Although the overall model approached but did not reach conventional significance levels, examination of individual predictors revealed that gender was a significant factor (β = -1.06, *p* = .013), with males scoring higher than females. Age (β = 0.02, *p* = .294) and country (β = 0.39, *p* = .405) were not significant predictors of total scores.

Analysis of the subscales showed distinct patterns. For the Catastrophic Thinking subscale, demographics explained 1.12% of the variance (R² = 0.011, F(3, 1655) = 6.24, *p* < .001), with gender again emerging as a significant predictor (β = -1.28, *p* < .001). For the Infection Worries subscale, which accounted for 0.5% of variance (R² = 0.005, F(3, 1655) = 2.77, *p* = .040), country was a significant predictor (β = -0.31, *p* = .020), with non-US participants scoring higher than US participants.

Despite these isolated significant effects, the consistently small proportion of variance explained (<1.2% across all scales) indicates that demographic characteristics have minimal practical influence on PHAID scores, supporting the measure's generalizability across diverse populations.

**Appendix S2: Conceptual Examination of Items Removed during EFA**

Following the Exploratory Factor Analysis (EFA) that established the factor structure using rigorous statistical criteria, we conducted a conceptual analysis of the removed items to complement the statistical findings, to better understand why these items were removed:

1. **Item 3 ("I aware of cough, fever, or breathing problem")**: This item measures general symptom awareness, which is a potential precursor to health anxiety but does not directly capture the affective (worry, fear) or cognitive (catastrophic misinterpretation) core of the construct. Its conceptual divergence from the scale's focus likely contributed to its low statistical loading.
2. **Item 4 ("I can control my thoughts on COVID-19")**: This construct is conceptually distinct from the scale's unified measurement of uncontrolled anxious thoughts and fears, likely explaining its statistical issue with cross-loadings.
3. **Items 15 & 17 ("If I have COVID-19, I would not be able to enjoy life" and "Having COVID-19 would ruin many aspects of my life")**: These items assess the catastrophic outcomes of being ill, whereas the retained items in Factor 1 measure the catastrophic process of health-related thinking. This conceptual difference, between fearing the results of an illness and engaging in illness-related thoughts, underpinned their statistical misfit and removal.

In conclusion, while the item removal was driven by statistical criteria, this conceptual review indicates that the final 12-item PHAID achieved greater conceptual coherence by retaining items that directly measure the core cognitions and emotions of infectious disease-related health anxiety.

Table S1

Participant characteristics

|  | **Number, mean (SD) or %** |
| --- | --- |
| **Time 1** | |
| *n* | 1660 |
| Age (years), mean | 36.94 (11.97) |
| Gender, % |  |
| Males | 49.5 |
| Females | 50.5 |
| Country, % |  |
| United States | 68.4 |
| India | 12.6 |
| Brazil | 7.0 |
| Canada | 4.0 |
| Italy | 2.3 |
| Other countries | 5.6 |
| Ethnicity‌, % |  |
| Non-Hispanic White | 53.5 |
| Black or African American | 7.7 |
| American Indian or Alaska Native | 2.7 |
| East Asian | 3.9 |
| Native Hawaiian or Other Pacific Islander | 0.2 |
| Hispanic/Latino | 9.2 |
| South Asian | 15.7 |
| Chinese | 0.8 |
| Arab | 0.6 |
| Jewish | 0.7 |
| Mixed | 2.7 |
| Other | 2.4 |
| Education‌, % |  |
| Primary school or below | 0.1 |
| Junior High school | 0.5 |
| High school | 18 |
| Associate's degree | 10.1 |
| Bachelor's degree | 50.9 |
| Graduate degree (master, professional, or doctoral degree) | 20.5 |
| Peking Health Anxiety Scale for Infectious Diseases |  |
| Total score | 25.91 (8.49) |
| Catastrophic Thinking | 17.54 (6.92) |
| Infection worries | 7.67 (2.43) |
| Hospital Anxiety and Depression Scale |  |
| Anxiety | 8.36 (4.52) |
| Depression | 8.42 (3.66) |
| Wash hands items |  |
| 1. In the past three days, as you arrived home, did you wash your hands with soap and running water or sanitize in time? | 4.32 (1.03) |
| 2. In the past three days, did you wash your hands thoroughly with soap and running water or sanitize in time before cooking and eating? | 4.32 (1.01) |
| 3. In the past three days, after each cough or sneeze, did you wash your hands thoroughly with soap and running water or sanitize in time? | 3.77 (1.24) |
| 4. In the past three days, after each visit to the restroom, did you wash your hands thoroughly with soap and running water or sanitize in time? | 3.77 (1.24) |
| **Time 2** | |
| *n* | 355 |
| Age (years), mean | 39.21 (12.69) |
| Gender, % |  |
| Males | 51.0 |
| Females | 49.0 |
| Country, % |  |
| United States | 62.5 |
| India | 16.3 |
| Brazil | 9.6 |
| Canada | 2.8 |
| Italy | 3.4 |
| Other countries | 5.4 |
| Ethnicity‌, % |  |
| Non-Hispanic White | 59.2 |
| Black or African American | 5.4 |
| American Indian or Alaska Native | 0.8 |
| East Asian | 3.4 |
| Native Hawaiian or Other Pacific Islander | 0.3 |
| Hispanic/Latino | 7.6 |
| South Asian | 17.2 |
| Chinese | 1.1 |
| Arab | 0 |
| Jewish | 0.3 |
| Mixed | 2.8 |
| Other | 2.0 |
| Education‌, % |  |
| Primary school or below | 0 |
| Junior High school | 0.6 |
| High school | 17.7 |
| Associate's degree | 12.4 |
| Bachelor's degree | 47.9 |
| Graduate degree (master, professional, or doctoral degree) | 21.4 |
| Peking Health Anxiety Scale for Infectious Diseases |  |
| Total score | 22.4 (8.02) |
| Catastrophic Thinking | 15.32 (6.01) |
| Infection worries | 7.10 (2.64) |

Table S2

Parallel analysis of eigenvalues

| **Root** | **Actual** | **Average** | **95^th^ Percentile** |
| --- | --- | --- | --- |
| 1 | 8.301 | 1.265 | 1.312 |
| **2** | **1.527** | **1.215** | **1.248** |
| 3 | 1.183 | 1.177 | 1.206 |
| 4 | 1.051 | 1.142 | 1.167 |
| 5 | 0.741 | 1.112 | 1.136 |
| 6 | 0.681 | 1.084 | 1.106 |
| 7 | 0.609 | 1.057 | 1.079 |
| 8 | 0.509 | 1.031 | 1.053 |
| 9 | 0.468 | 1.007 | 1.028 |
| 10 | 0.419 | 0.982 | 1.003 |
| 11 | 0.405 | 0.957 | 0.978 |
| 12 | 0.366 | 0.933 | 0.954 |
| 13 | 0.332 | 0.908 | 0.929 |
| 14 | 0.323 | 0.883 | 0.904 |
| 15 | 0.300 | 0.857 | 0.880 |
| 16 | 0.281 | 0.829 | 0.854 |
| 17 | 0.272 | 0.799 | 0.825 |
| 18 | 0.231 | 0.762 | 0.793 |

*Note.* The eigenvalue of actual data and parallel random data for adolescents. Actual = eigenvalue of actual data; Average = average eigenvalue of parallel random data; 95^th^ Percentile = 95^th^ percentile eigenvalue of parallel random data.

Table S3

Candidate cutoff scores of the PHAID with corresponding sensitivity, specificity, and Youden's index

| **Cutoff score** | **Sensitivity** | **Specificity** | **Youden index** | **Cutoff score** | **Sensitivity** | **Specificity** | **Youden index** |
| --- | --- | --- | --- | --- | --- | --- | --- |
| **11.0** | 1.000 | 0.000 | 0.000 | **30.50** | 0.447 | 0.952 | 0.399 |
| **12.5** | 0.983 | 0.078 | 0.062 | **31.50** | 0.406 | 0.958 | 0.364 |
| **13.5** | 0.975 | 0.123 | 0.098 | **32.50** | 0.357 | 0.962 | 0.319 |
| **14.5** | 0.963 | 0.197 | 0.160 | **33.50** | 0.313 | 0.971 | 0.284 |
| **15.5** | 0.943 | 0.288 | 0.231 | **34.50** | 0.259 | 0.974 | 0.233 |
| **16.5** | 0.926 | 0.373 | 0.299 | **35.50** | 0.227 | 0.978 | 0.205 |
| **17.5** | 0.912 | 0.440 | 0.352 | **36.50** | 0.187 | 0.981 | 0.168 |
| **18.5** | 0.895 | 0.495 | 0.390 | **37.50** | 0.153 | 0.984 | 0.137 |
| **19.5** | 0.874 | 0.566 | 0.440 | **38.50** | 0.130 | 0.987 | 0.117 |
| **20.5** | 0.851 | 0.615 | 0.466 | **39.50** | 0.105 | 0.991 | 0.097 |
| **21.5** | 0.818 | 0.677 | 0.496 | **40.50** | 0.082 | 0.994 | 0.076 |
| **22.5** | 0.791 | 0.744 | 0.534 | **41.50** | 0.064 | 0.994 | 0.058 |
| **23.5** | **0.760** | **0.790** | **0.550** | **42.50** | 0.041 | 0.996 | 0.037 |
| **24.5** | 0.714 | 0.834 | 0.548 | **43.50** | 0.030 | 0.996 | 0.026 |
| **25.5** | 0.673 | 0.861 | 0.534 | **44.50** | 0.019 | 0.996 | 0.014 |
| **26.5** | 0.637 | 0.887 | 0.524 | **45.50** | 0.013 | 0.997 | 0.011 |
| **27.5** | 0.603 | 0.902 | 0.504 | **46.50** | 0.010 | 0.997 | 0.007 |
| **28.5** | 0.559 | 0.923 | 0.483 | **47.50** | 0.010 | 0.999 | 0.009 |
| **29.5** | 0.512 | 0.939 | 0.451 | **49.00** | 0.000 | 1.000 | 0.000 |

Table S4

Discrimination and difficulty parameters for scale items

| Model | Item | a | b1 | b2 | b3 |
| --- | --- | --- | --- | --- | --- |
| Catastrophic thinking | Item 9 | 3.208 | -0.08 | 0.74 | 1.63 |
|  | Item 13 | 2.887 | -0.39 | 0.66 | 1.72 |
|  | Item 7 | 2.87 | -0.43 | 0.65 | 1.63 |
| Infectious worry | Item 5 | 3.68 | -1.39 | -0.03 | 0.98 |
|  | Item 1 | 3.384 | -1.67 | -0.16 | 0.96 |

*Note.* a: discrimination parameters; b: difficulty parameters.

Table S5

Candidate cutoff scores of the PHAID-S with corresponding sensitivity, specificity, and Youden's index

| **Cutoff score** | **Sensitivity** | **Specificity** | **Youden index** |
| --- | --- | --- | --- |
| 4.0 | 1.000 | 0.000 | 0.000 |
| 5.5 | 0.981 | 0.100 | 0.081 |
| 6.5 | 0.968 | 0.168 | 0.136 |
| 7.5 | 0.928 | 0.372 | 0.300 |
| 8.5 | 0.883 | 0.509 | 0.393 |
| 9.5 | 0.831 | 0.634 | 0.465 |
| **10.5** | **0.727** | **0.744** | **0.470** |
| 11.5 | 0.653 | 0.845 | 0.498 |
| 12.5 | 0.547 | 0.909 | 0.456 |
| 13.5 | 0.426 | 0.942 | 0.368 |
| 14.5 | 0.324 | 0.965 | 0.289 |
| 15.5 | 0.208 | 0.978 | 0.187 |
| 16.5 | 0.118 | 0.988 | 0.106 |
| 17.5 | 0.067 | 0.993 | 0.060 |
| 18.5 | 0.039 | 0.994 | 0.033 |
| 19.5 | 0.028 | 0.994 | 0.022 |
| 21.0 | 0.000 | 1.000 | 0.000 |

Figure S1

Test information curves (TIC) and item information curves (IIC) and for the catastrophic thinking subscale for PHAID.


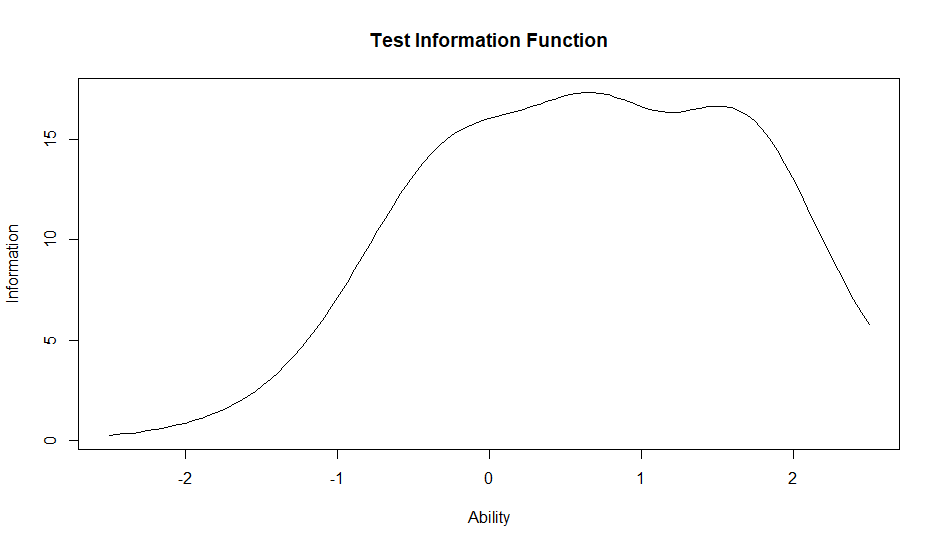


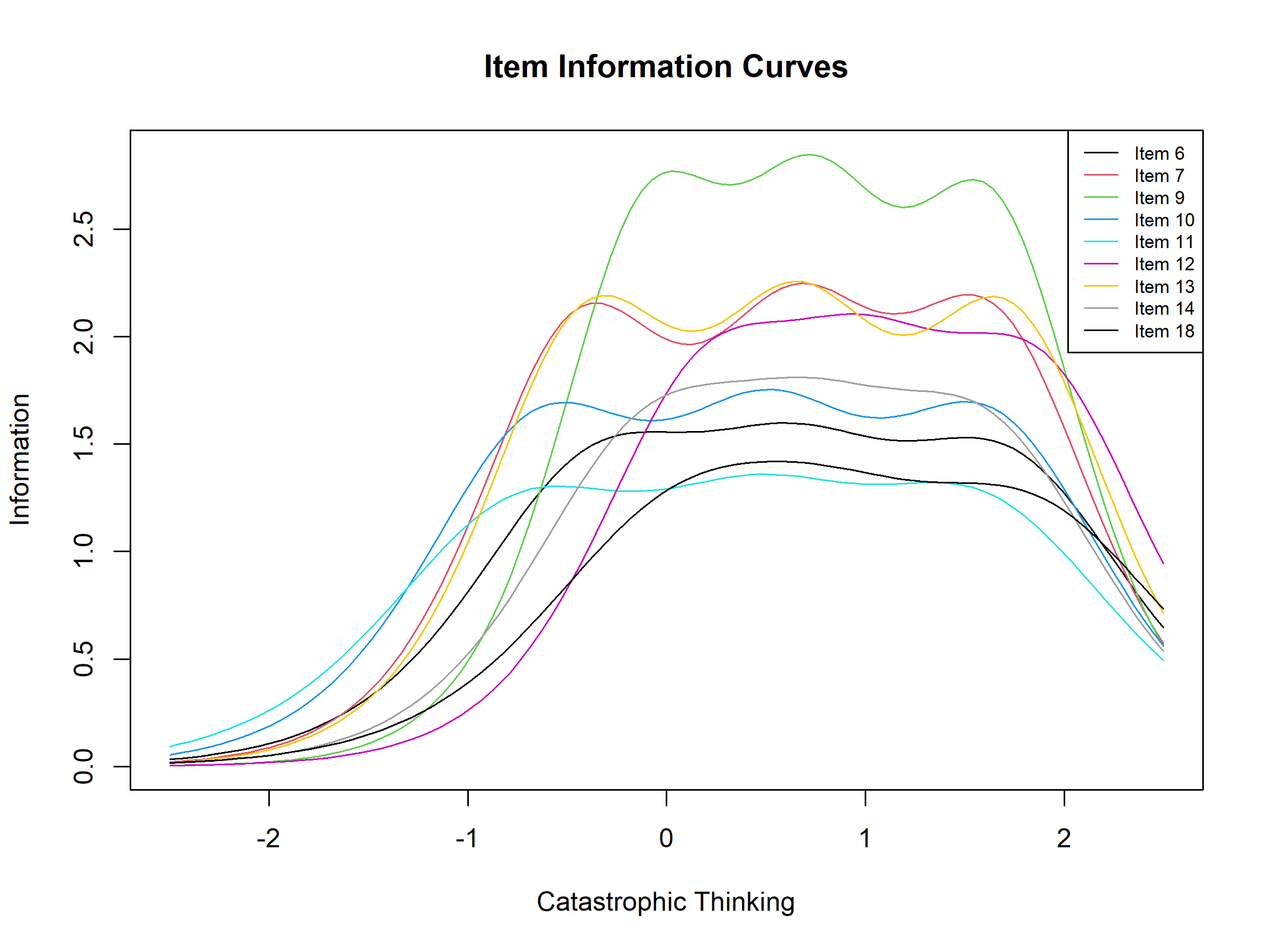


Figure S2

Test information curves (TIC) and item information curves (IIC) and for the infectious worry subscale for PHAID.


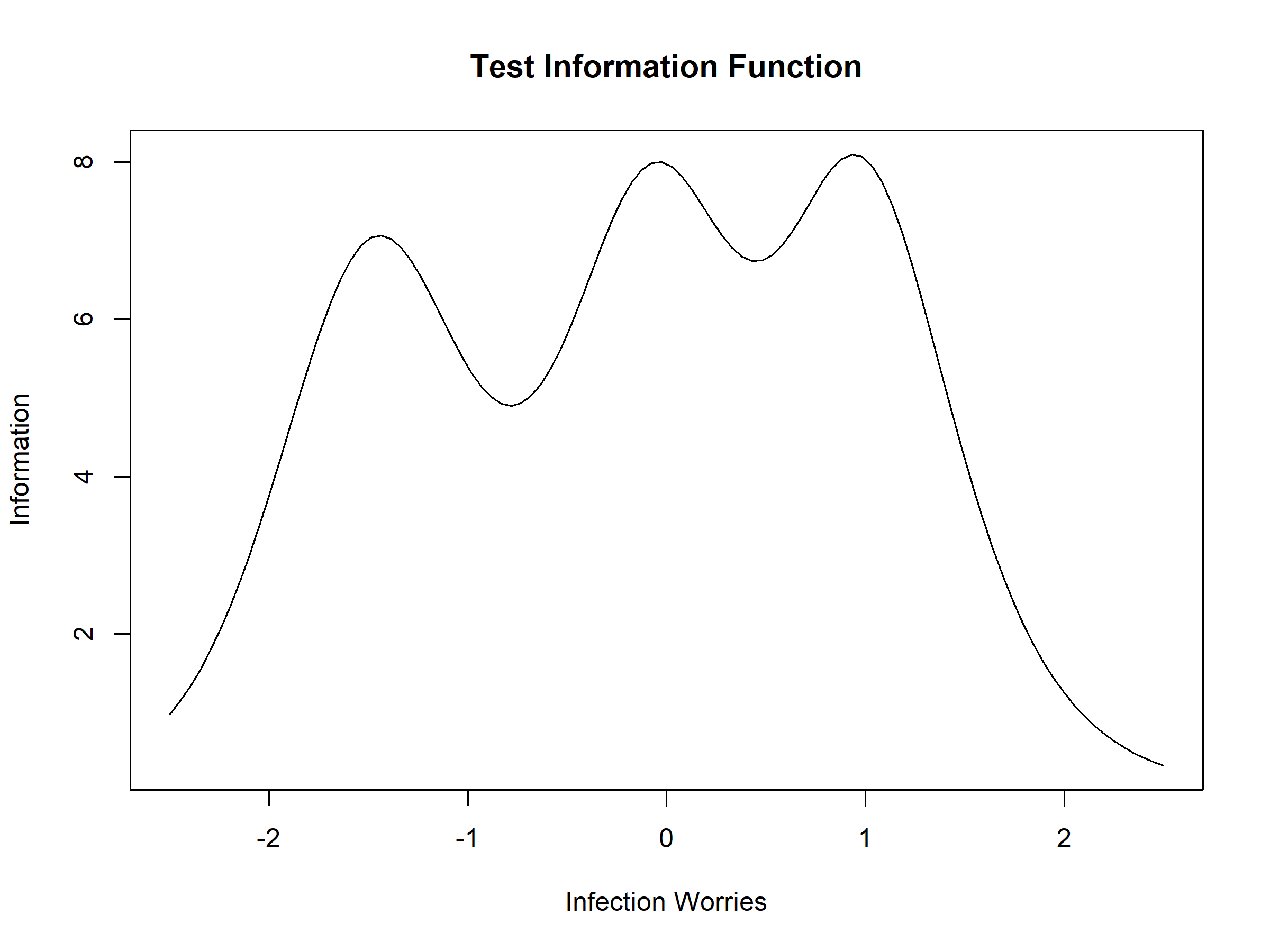


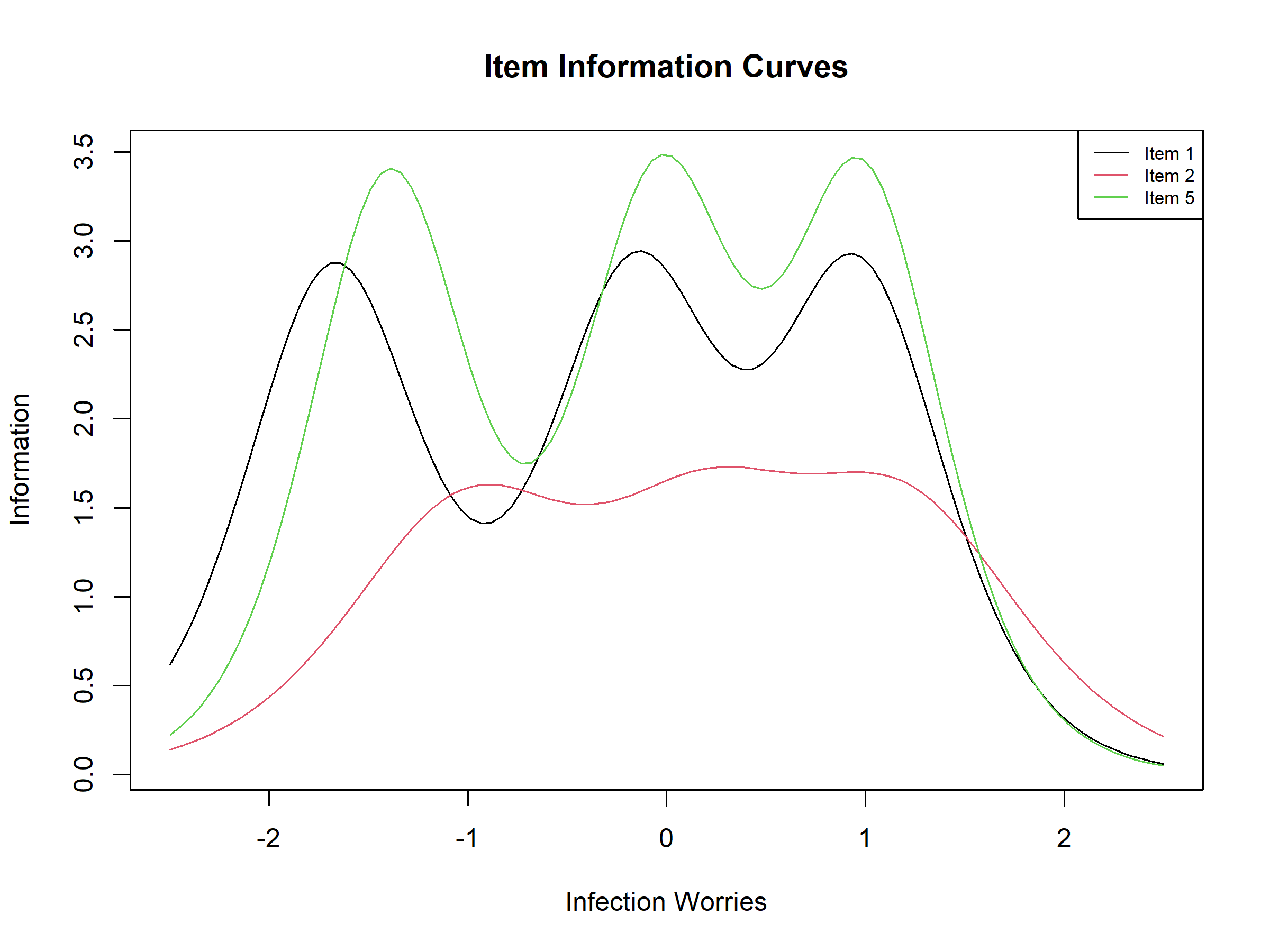


Figure S3

ROC curves for PHAID-S.


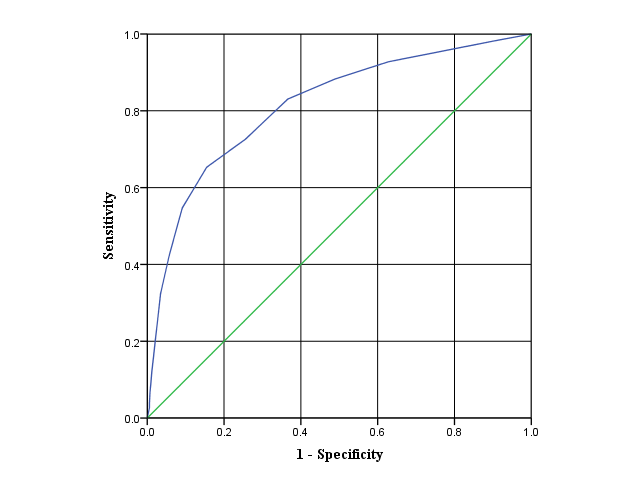


*Note.* PHAID-S = Peking Health Anxiety Scale for Infectious Diseases short form.

Figure S4

Test information curves (TIC) and item information curves (IIC) and for the catastrophic thinking subscale for PHAID-S.


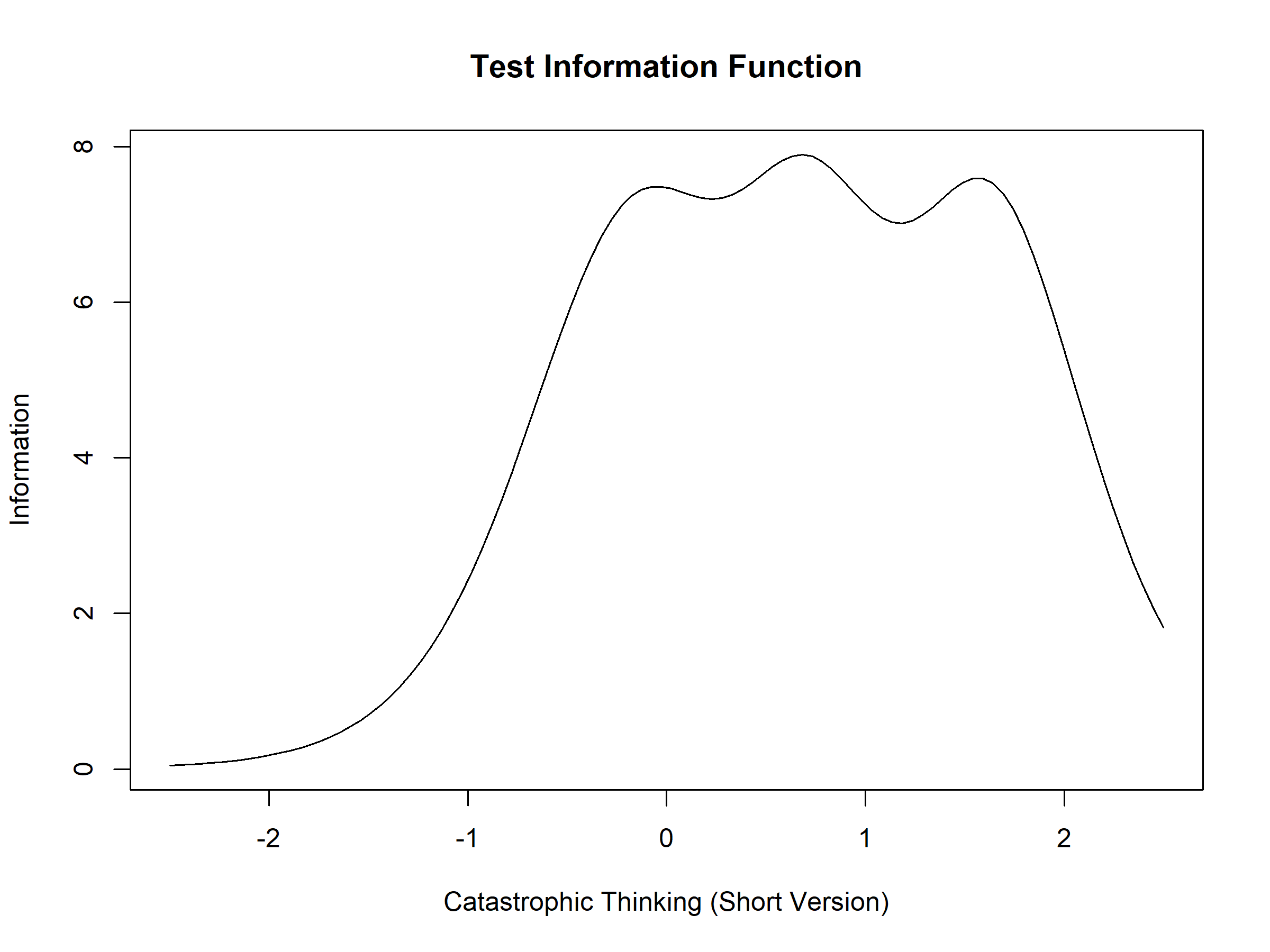


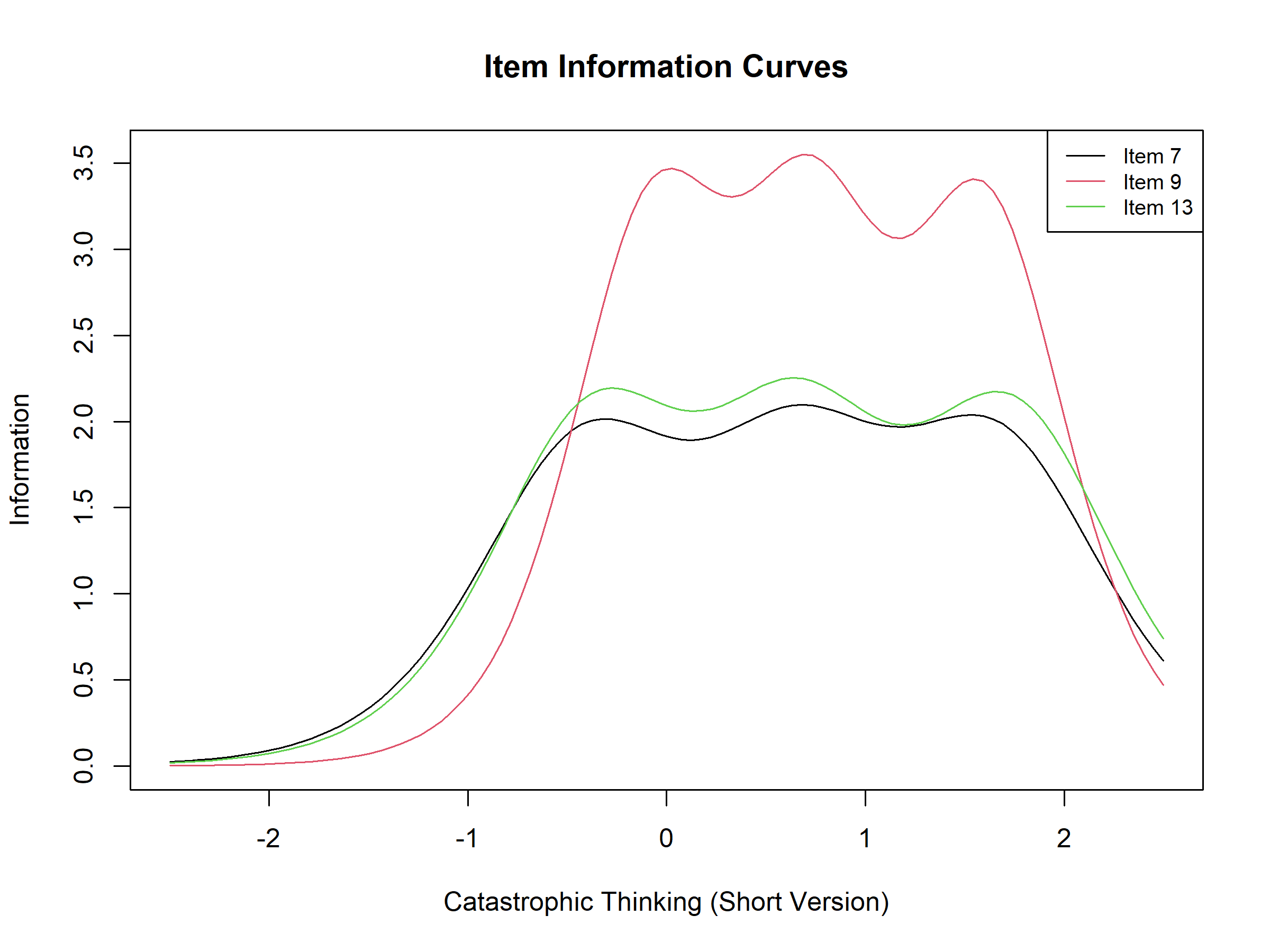


Figure S5

Test information curves (TIC) and item information curves (IIC) and for the infectious worry subscale for PHAID-S.


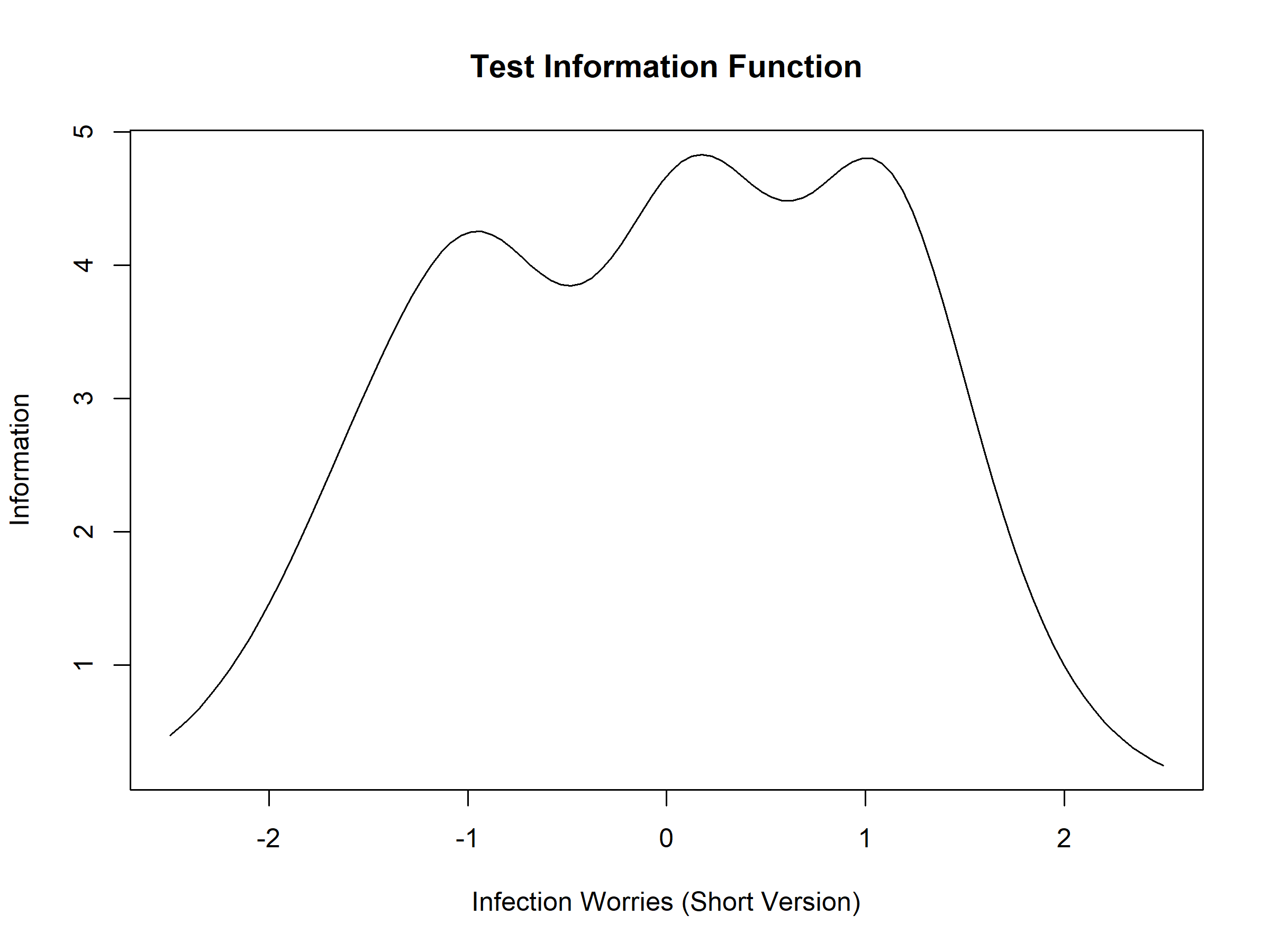


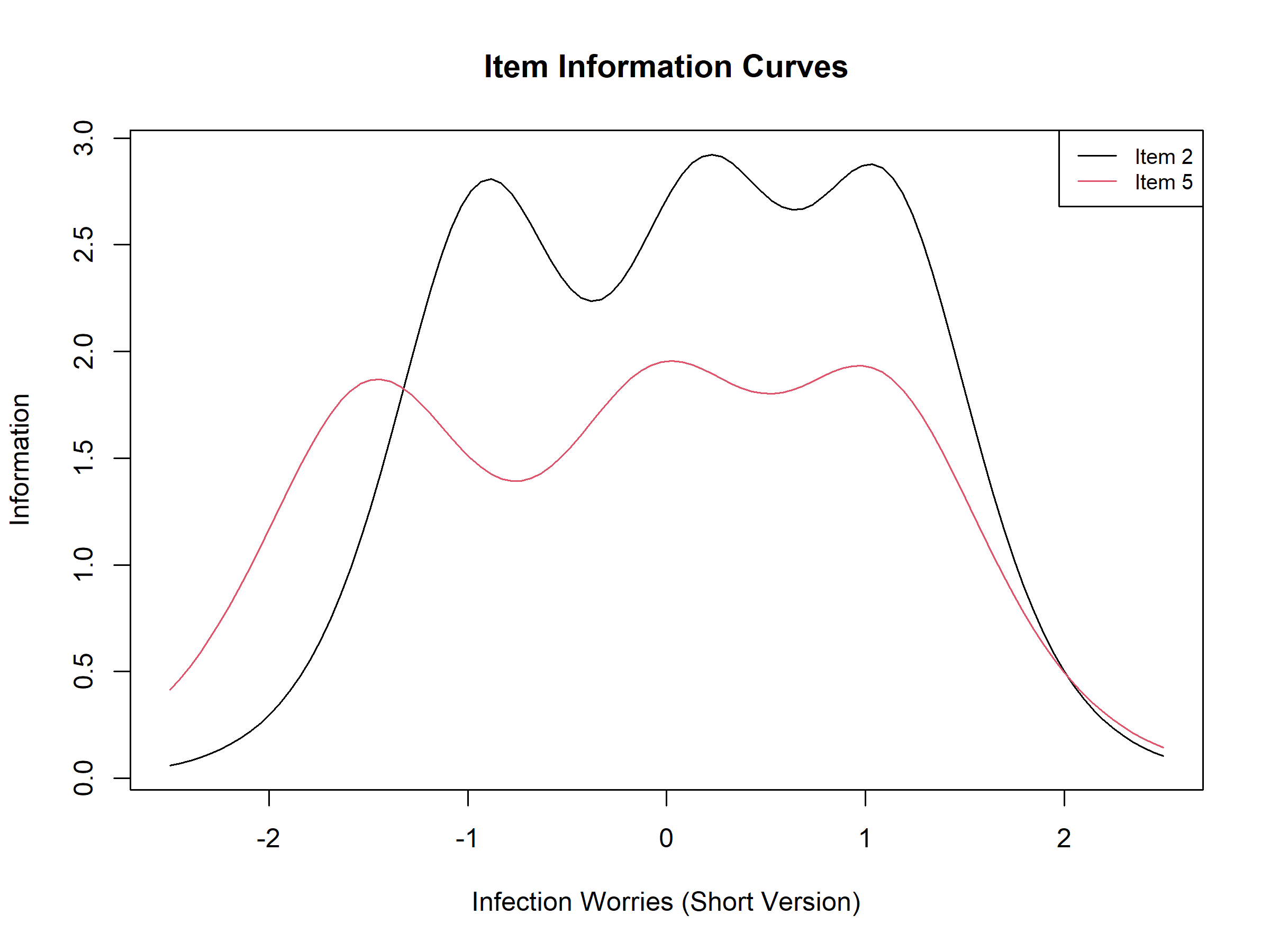

Supplement: Supplementary file 1 [file SupplementaryFile1.docx]
